# Mortality in Inflammatory Rheumatic Diseases: Lithuanian National Registry Data and Systematic Review

**DOI:** 10.3390/ijerph182312338

**Published:** 2021-11-24

**Authors:** Jolanta Dadonienė, Greta Charukevič, Gabija Jasionytė, Karolina Staškuvienė, Dalia Miltinienė

**Affiliations:** 1 State Research Institute Centre for Innovative Medicine, LT-08406 Vilnius, Lithuania; jolanta.dadoniene@mf.vu.lt; 2 Department of Public Health, Institute of Health Sciences, Vilnius University Faculty of Medicine, LT-03101 Vilnius, Lithuania; greta.charukevic@mf.stud.vu.lt; 3 Clinic of Rheumatology, Orthopaedics Traumatology and Reconstructive Surgery, Institute of Clinical Medicine, Vilnius University Faculty of Medicine, LT-03101 Vilnius, Lithuania; gabija.jasionyte@gmail.com (G.J.); k.sukackaite1994@gmail.com (K.S.)

**Keywords:** rheumatic diseases, mortality, standardized mortality ratio, life expectancy, systematic review

## Abstract

Despite significant improvement in survival, rheumatic diseases (RD) are associated with premature mortality rates comparable to cardiovascular and neoplastic disorders. The aim of our study was to assess mortality, causes of death, and life expectancy in an inflammatory RD retrospective cohort and compare those with the general population as well as with the results of previously published studies in a systematic literature review. Patients with the first-time diagnosis of inflammatory RD during 2012–2019 were identified and cross-checked for their vital status and the date of death. Sex- and age-standardized mortality ratios (SMR) as well as life expectancy for patients with inflammatory RDs were calculated. The results of a systematic literature review were included in meta-standardized mortality ratio calculations. 11,636 patients with newly diagnosed RD were identified. During a total of 43,064.34 person-years of follow-up, 950 death cases occurred. The prevailing causes of death for the total cohort were cardiovascular diseases and neoplasms. The age- and sex-adjusted SMR for the total cohort was calculated to be 1.32 (1.23; 1.40). Patients with rheumatoid arthritis if diagnosed at age 18–19 tend to live for 1.63 years less than the general population, patients with spondyloarthritis—for 2.7 years less, patients with connective tissue diseases—for almost nine years less than the general population. The findings of our study support the hypothesis that patients with RD have a higher risk of mortality and lower life expectancy than the general population.

## 1. Introduction

Although rheumatic diseases (RD) are generally regarded as non-fatal diseases, some of them are associated with premature mortality rates comparable to cardiovascular and neoplastic disorders. Significant improvement in survival has been noted since the introduction of modern cytotoxic therapies into the treatment of inflammatory RD, but it is still estimated, that mortality rates for patients with inflammatory RD are 59–425% higher than those of the age- and sex-matched general population. The mortality rate is the highest for systemic connective tissue diseases (CTD) (such as systemic lupus erythematosus (SLE), systemic sclerosis (SSc)) and systemic vasculitis (SV) patients (standardized mortality ratio (SMR) up to 5.25), and the loss in life expectancy at the time of birth compared with the general population is the greatest for female patients with SSc (34 years). Rheumatoid arthritis (RA) and spondyloarthritis (SpA) patients have a less elevated risk for premature death (SMR 1.3–3.0), the loss in life expectancy is 5 to 7 years [1,2,3,4,5]. 

Infection remains the most common cause of death in inflammatory RD as a result of immunosuppressive treatment and changes in the immune system caused by the disease itself [2]. Chronic inflammation present in inflammatory RD is associated with an increased risk of atherosclerosis and arterial thrombosis. The standardized incidence ratios of coronary heart disease and cerebrovascular disease are increased in patients with RA, SLE, ankylosing spondylitis (AS), and psoriatic arthritis (PsA). These cardiovascular complications are major causes of death, second to infection [2,6,7,8,9,10]. Organ dysfunction as a result of the disease itself, such as renal failure in patients with SLE or interstitial lung fibrosis with pulmonary hypertension in SSc, is another major cause of reduced survival in RDs [2].

To the best of our knowledge, no study has addressed the impact of inflammatory RD on life expectancy and premature mortality in Lithuania or neighboring Eastern European countries.

Therefore, the aim of our study was to assess mortality, causes of death, and life expectancy in an inflammatory RD retrospective cohort and compare those with the general population as well as with the results of previously published studies. 

## 2. Materials and Methods

### 2.1. Data Sources

After obtaining the approval from Vilnius Regional Bioethics Committee (approval number 158200-17-958-462), the study was performed using the data of the Lithuanian Compulsory Health Insurance Information System database SVEIDRA. It is a population-based database with the data collected from 1995; although computerized data eligible for research are only available from 2005. The data captures all physician visits, procedures, hospitalizations, diagnoses, and prescribed reimbursed medications to all residents of Lithuania. The information sources are health care institutions (both state-run and private) and medication prescriptions released by pharmacies. 

We have requested the information from SVEIDRA on all patients who had a first-time diagnosis of inflammatory RD between 1 January 2012 and 31 December 2019. These rheumatologic conditions included rheumatoid arthritis (diagnosis codes M05 and M06 according to International Classification of Diseases 10th version (ICD-10), psoriatic arthritis (M07), systemic connective tissue diseases and vasculitis (M30–35), ankylosing spondylitis and spondyloarthritis (M45, M46). We have also requested information about prescription of glucocorticoids (prednisolone or methylprednisolone), conventional synthetic disease-modifying anti-rheumatic drugs (csDMARDs) (methotrexate, azathioprine, leflunomide, sulfasalazine, hydroxychloroquine), or biological disease-modifying anti-rheumatic drugs (bDMARDs) (infliximab, etanercept, adalimumab, tocilizumab, or rituximab with available biosimilars).

In total, 95,289 RD cases, first time diagnosed between 2012 and 2019, were selected. At least one year of no data about RD before the index date was required to exclude prevalent cases. We excluded 22,526 cases primarily diagnosed in 2012 as it was impossible to verify their RD diagnosis before 2012 because no data preceding that year was available.

**Figure 1:**
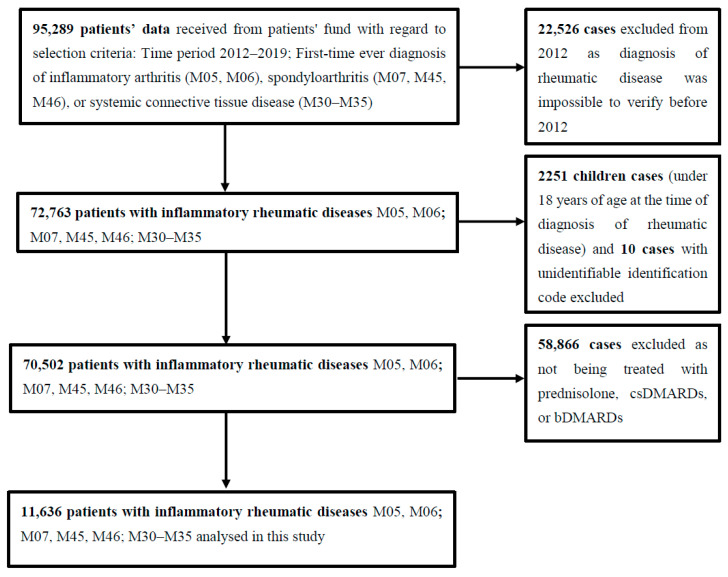
Details of study methodology.

2251 cases of children (<18 years old at the time of diagnosis) were excluded as well as 10 cases with unidentifiable identification codes. Participants were classified as cases if they had records of at least one prescription of the medications for RD reimbursed by the state. 58,866 cases with no information about prescribed reimbursed treatment with glucocorticoids, csDMARDs, or bDMARDs were excluded. Finally, 11,636 cases were included in the final analysis as is demonstrated in Figure 1.

The final 11,636 cases were cross-checked with the Health Information Center at the Institute of Hygiene, for vital status, date and cause of death if the fact of death was documented. The personal identification code was used for cross-checking the cases.

Available data for the final analysis included sex, age, ICD-10 code of RD, date of diagnosis of RD, date of death, cause of death, and information about the state’s reimbursement for the prescribed drugs.

For comparison with national estimates, the information on the adult Lithuanian population census in 2013–2019 was obtained from *Statistics Lithuania* (www.stat.gov.lt, (accessed on 8 January 2021)).

### 2.2. Systematic Literature Review and Meta-Analysis 

We have adopted the recommendations of the Cochrane Collaboration [11] and the Preferred Reporting Items for Systematic Reviews and Meta-Analyses (PRISMA) statement [12] for conducting the systematic review and reporting its results.

**Literature search**. PubMed, OVID, EBSCO, Cochrane Library, ScienceDirect, Taylor & Francis, and SpringerLink were searched using the term “standardized mortality” with each of the following diagnoses: “rheumatoid arthritis”, “psoriatic arthritis”, “ankylosing spondylitis”, “non-radiographic axial spondyloarthritis”, “enteropathic arthritis”, “systemic lupus erythematosus”, “mixed connective tissue disease”, “Sjogren’s syndrome”, “systemic sclerosis”, “microscopic polyangiitis“, “granulomatosis with polyangiitis“, “eosinophilic granulomatosis with polyangiitis“, “giant cell arteritis“, “polyarteritis nodosa“, “Takayasu arteritis“, “panniculitis“, “Behcet‘s disease“, “dermatomyositis”, “polymyositis”, and “polymyalgia rheumatica”. The keyword “standardized mortality” was used instead of the full term (“standardized mortality ratio”) to decrease the chance of missing any significant studies. We have also looked through the references of all retrieved articles for other relevant publications. The last date of the search was 28 February 2021. 

**Eligibility criteria**. The inclusion criteria were: (1) prospective and retrospective cohort trials, (2) study population with one of the previously listed RDs, (3) SMR of specific RD is reported or can be calculated, (4) studies published between 1 January 2010 and 31 December 2020 in English or Lithuanian languages. We have excluded the studies: (1) including only children (younger than 15 years), (2) reporting only hypothetical empirical data, (3) reporting SMRs but the etiology of mortality is not related with the rheumatic condition, (4) with no full text available, (5) with duplicate (overlapping) patient populations.

**Screening and data extraction**. Three reviewers performed the literature search and screening independently. Any disagreements among reviewers were discussed until consensus was reached. After the exclusion of duplicates, studies published before 2010, and articles without full text available, the reviewers analyzed the titles and abstracts of retrieved articles. The full texts of potentially relevant studies were assessed for eligibility. The following data were extracted from the studies and included in the final analysis: the authors, year of publication, number of patients, number of deaths during the study period, and SMRs.

### 2.3. Statistical Methods

The life expectancy and mortality in the study population were assessed in a retrospective cohort study. Person-years of follow-up were calculated from the date of RD diagnosis to the first date of one of the following events: death or the end of follow-up (31 December 2019). Sex- and age-SMRs were calculated by dividing the observed number of deaths among rheumatic patients by the expected number of deaths; the latter was calculated using national rates from the Lithuanian Department of Statistics Official Statistics Website; 95% confidence intervals (CIs) for SMRs were calculated as well. 

Life expectancy for patients with inflammatory RD was estimated by standard single-decrement life-table analysis as described by Perron L. [13].

The principal causes of death, as coded by the attending physician or family doctor in the death registry of the Health Information Center at the Institute of Hygiene, were obtained for patients with the inflammatory RD who died between 2012 and 2019. These were grouped under the following categories: infections, malignancies, cardiovascular and circulatory complications, cerebrovascular complications, respiratory causes (excluding infections), renal failure, trauma and poisoning (including suicide), and related to RD activity but with unspecified exact causes.

All statistical calculations for this retrospective study were carried out using Microsoft Excel 2016 (Microsoft Corporation, St. Redmond, WA, USA) spreadsheet and tables. The meta-standardized mortality ratio was calculated using the free available *WinPepi* statistical package with a meta-analysis calculator (Version 11.65, copyright Abramson JH, 23 August 2016) for ratios.

## 3. Results

**Demographic characteristics of patients in the retrospective cohort study**. During the period between 2013–2019 we have identified 11636 patients with RD (6008 patients with RA, 3289 with SpA (including PsA), and 2339 with systemic CTD and vasculitis). The mean duration of follow-up was 3.70 years. The cohort consisted mainly of women (70%), especially in RA and systemic CTD groups (77% and 76%). Sex distribution in the SpA group was roughly equal (52% of women and 48% of men). Half of the total cohort were RA patients (52%). The mean age of the patients at the time of RD diagnosis was 57 years (range 18–97). The main characteristics of the patients included in the final analysis are provided in Table 1.

**Main causes of death**. During a total of 43,064.34 person-years of follow-up, 950 death cases occurred between 2013–2019 and half of them (53.5%) were due to underlying RA. At the time of death, RA patients tend to be the oldest (74.95 years (11.45)), following the CTD (73.98 years (12.37)) and SpA (67.34 years (13.45)) groups.

The prevailing causes of death for the total cohort were cardiovascular diseases, neoplasms including lymphoproliferative malignancies, and less often diseases of the respiratory system. Cardiovascular diseases and neoplasms remain the main cause of death in all three groups of RD and they make up to 70 percent of all the causes of death. The third most common cause of death differs among the subtypes. In RA and CTD groups the diseases of the respiratory system are in the third place while for SpA patients the third place is taken by external causes of death and reflect the distribution of the causes of death in the general population. 

Musculoskeletal diseases as a primary cause of death are of some importance in the systemic CTD group while generally it has no major impact. The main causes of death in descending order are presented in Table 2.

**Standardized mortality ratios**. The age- and sex-adjusted SMR for the total cohort was calculated to be 1.32 (1.23; 1.40) and was similar in men and women (1.32 (1.19; 1.46) and 1.31 (1.21; 1.42), respectively). SMR was the highest in the CTD diseases group (1.55 (1.38; 1.73)), followed by the RA group (1.25 (1.14; 1.36)). Mortality in the SpA group, including PsA and AS, was calculated to be the most similar to the general population (SMR 1.16 (0.98; 1.37). SMRs of particular RD are presented in Table 3. 

**Figure 2:**
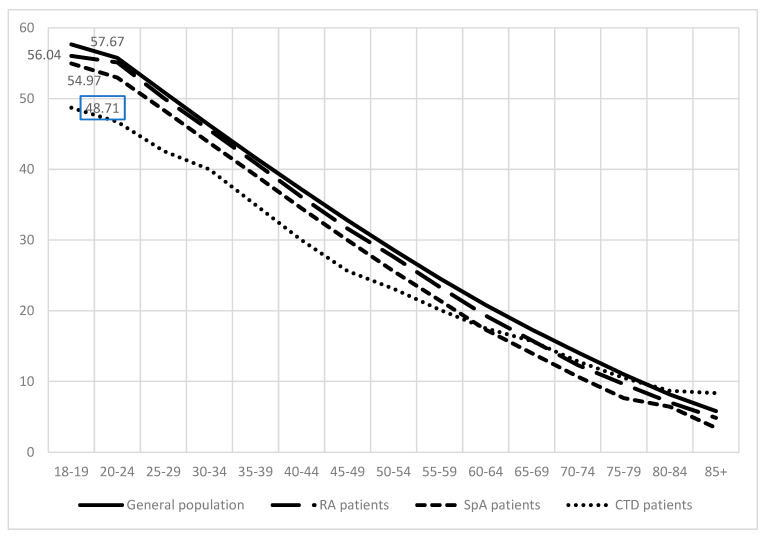
Life expectancy of general population, rheumatoid arthritis (RA) patients, spondyloarthritis (SpA) (including psoriatic arthritis and ankylosing spondylitis), and connective tissue diseases (CTD) patients.

**Life expectancy of rheumatic patients**. According to population census data, the life expectancy of the general population from age 18–19 in 2019 was 57.67 years. RA patients if diagnosed at age 18–19 tend to live for 1.63 years less than the general population, followed by SpA patients who live for 2.7 years less than the general population (Figure 2).

Patients with CTD tend to live the shortest. Their life expectancy was reduced by almost 9 years if compared to the general population.

**Literature search results**. 15 studies on SLE, 12 on RA, 12 on SV, 10 on SSc, 5 on PsA, 4 on Sjogren’s syndrome, 4 on dermatomyositis and polymyositis, 3 on AS, and one study on polymyalgia rheumatica were included in the final review and meta-analysis. Overall, this resulted in 55 studies since some authors reported SMRs for a few RD [2,14,15]. There were no studies found on mixed connective tissue disease, non-radiographic axial spondyloarthritis, enteropathic arthritis, panniculitis, eosinophilic granulomatosis with polyangiitis and polyarteritis nodosa meeting the complete set of our criteria.

Flowcharts of every search and the table summarizing the results of included studies are presented in Supplementary Materials.

**Meta-analysis of standardized mortality ratios between different studies and disease entities**. The results of the meta-analysis of SMRs among eligible studies (including our study) are presented in Table 4. The highest excess mortality was demonstrated for the myositis group following SLE and SSc. Polymyalgia rheumatica and PsA showed the lowest meta-SMR if compared to other diseases. The inconsistency of results among different studies is notable, however.

## 4. Discussion

To our knowledge, this is the first study conducted in Lithuania and in Eastern Europe designed to evaluate mortality statistics in a broad spectrum of inflammatory RD. We have found a significant increase in the mortality rate in the total cohort of inflammatory RD patients compared with the general Lithuanian population. The results we have presented respond well to the worldwide estimations of SMR. Age- and sex-adjusted SMR for the total Lithuanian cohort was calculated to be 1.32. The highest SMR was calculated for vasculitis and myositis patients (SMR 3.24, both equally), followed by SSC and SLE patients (2.66 and 2.53, respectively). Mortality of RA and polymyalgia rheumatica patients only slightly exceeded mortality observed in the general population (SMR 1.25 and 1.29, respectively), whereas AS and PsA patients‘ mortality did not significantly differ from the general population (SMR 1.25 and 1.04). We have also calculated the life expectancy of the Lithuanian RD cohort and confirmed the assumption that the CTD patients’ group tend to live the shortest life as their life expectancy was 9 years shorter compared to the general population. Cardiovascular diseases and neoplasms were dominating causes of death among RD patients together making up to 70 percent of all causes of death. 

Our study is one of the few addressing a broad spectrum of inflammatory RD [2,5,14,15], and the first one from the Eastern European geographical region. Usually, studies focus on the mortality of one group of RD patients, and the whole burden may be underrepresented. Mortality of systemic CTDs (especially SLE and SSc) [2,14,15,32,33,34,35,36,37,38,39,40,41,42,43,44,47,48,49,50,51,52,67,68,69,70,71,72,73] and SV [2,14,15,53,54,55,56,57,58,59,60,61,74,75,76] are covered better, as well as mortality of patients with RA and PsA [2,14,16,17,18,19,20,21,22,23,24,25,26,27,28,29,77,78,79,80,81,82,83,84,85,86,87,88,89,90]. The data on myositis, Sjogren’s syndrome, and especially AS and polymyalgia rheumatica mortality are still scarce and fragmented [2,14,15,30,31,45,46,62,63,64,65,66,77,91]. Myositis, SSc, SLE, and vasculitis are the diseases with the highest reported SMRs (ranging from 2.4 to 9.0 for myositis, from 1.34 to 5.7 for SSc, from 1.48 to 5.25 for SLE, and from 0.5 to 3.7 for vasculitis) [2,14,15,32,33,34,35,36,37,38,39,40,41,42,43,44,47,48,49,50,51,52,53,54,55,56,57,58,59,60,61,62,63,64,65]. SMRs for RA ranged from 1.04 to 1.89, for PsA—from 0.82 to 1.59, for AS—from 0.72 to 1.87, for Sjogren’s syndrome—from 1.1 to 2.11. Only one study reported SMR for polymyalgia rheumatica to be 0.70 [2,14,15,16,17,18,19,20,21,22,23,24,25,26,27,28,29,30,31,45,46,66]. After plotting our data into meta-analysis of SMRs among the studies from the last decade, we concluded that the highest observed mortality was demonstrated for the patients with myositis (meta-SMR 5.06), followed by SLE and SSc patients (2.65 and 2.55, respectively). The lowest meta-SMR (0.95) was calculated for polymyalgia rheumatica patients. Despite the profound heterogeneity of different studies, generally, the results of meta-analysis of SMRs are in accordance with the results of our study. Some discrepancies should be mentioned. 

The most inconsistent result was noted in the SV group—in our study, we have calculated the SMR of 3.24 (2.59; 4.01), while the meta-SMR was calculated to be 1.92 (1.44; 2.57). The explanation for that could be the rather small number of SV patients in our study and the possibility of using different definitions for this diverse disease entity in different countries. 

Another inconsistency came along when analyzing the causes of death in our cohort. Infections are usually reported to be among the most common causes of death of patients with RD, together with cardiovascular diseases, cancer, and fatal disease manifestations [1,2,3,15,31,32,34,36,37,38,40,43,44,50,53,58,61,63,64]. In our study, diseases of the cardiovascular system were acknowledged to be the most prevalent cause of death, followed by neoplasms. Infections were not among the top 5 reasons for death, registered for Lithuanian patients with RD. The underestimation of the impact of infections on the mortality of patients with RD could be explained by the hypothesis that attending physicians tend to code the most severe underlying concomitant chronic disease as a primary cause of death.

The main strength of our study comes from the statement that we have retrieved data referring to the entire Lithuanian population from a reliable source of information that follows EU regulations for data collection from every health care institution and pharmacy in Lithuania. Therefore, we could omit the bias arising from hospital or tertiary center-based registries of including only severe cases of inflammatory RD.

In choosing this database as a main source of information, we could not overcome several limitations. First, as it was a prescription-based study and one of the inclusion criteria was availability of information about the treatment with state-reimbursed medications, some cases of RD might be missed in a case when patient’s medications are not reimbursed by the state. Spondyloarthritis may serve as an example because the treatment of this disease is still poorly reimbursed by the state.

The second limitation of our study is the short duration of the retrospective follow-up. The mean follow-up period was 3.8 years, and this might be too short to show the effect of RD on mortality rates. The earlier data was inaccessible to us as we were not allowed to retrieve the data before 2012 by the existing law. 

We acknowledge that presented mortality data represent the pre-pandemic period of time and not the most updated situation, and hope that it will serve for validated comparative data for future studies for RD patients.

## 5. Conclusions

This is the first nationwide cohort study to assess mortality, life expectancy, and causes of death in the inflammatory RD retrospective cohort. Our findings support the hypothesis that patients with RD have a higher risk of mortality and lower life expectancy than the general population. The results revealed a 32% excess risk of death among patients with RD compared to the general Lithuanian population. Analysis of the causes of death of patients with inflammatory RD revealed that the two main causes of death are cardiovascular diseases and neoplasms, together making up to 70 percent of all causes of death. The results of the meta-analysis of standardized mortality ratios among eligible studies (including our study) showed the highest excess mortality rate in the myositis group of patients following systemic lupus erythematosus and systemic sclerosis.

Though limited by a short follow-up period, this study supports results published in the last decade and indicates the need for future follow-up of the cohort of RD patients in relation to pandemic circumstances.

## Figures and Tables

**Table 1:** Study group characteristics of incident cases of rheumatic diseases during the period 2013–2019.

Characteristic	Rheumatoid Arthritis (*n* = 6008)	Spondyloarthritis (*n* = 3289)	Systemic Connective Tissue Diseases (*n* = 2339)	Total (*n* = 11,636)
Female no, %	4613 (76.78)	1712 (52.05)	1787 (76.40)	8112 (69.71)
Male no, %	1395 (23.22)	1577 (47.94)	552 (23.60)	3524 (30.29)
Mean age at diagnosis of rheumatic disease (SD)	58.91 (15.01)	48.91 (14.42)	61.37 (17.22)	56.57 (16.08)
Mean years of follow-up (SD)	3.80 (2.04)	3.74 (2.0)	3.38 (2.10)	3.70 (2.05)
Total person years of follow-up	22,861.11	12,303.61	7899.61	43,064.34

**Table 2:** Main causes of death in patients with underlying rheumatic diseases.

Causes of Death	All Death Cases *n* = 950	Rheumatoid Arthritis Death Cases *n* = 509	Spondyloarthropathies Death Cases *n* = 142	Systemic Connective Tissue Diseases Death Cases *n* = 299
Diseases of the circulatory system no, (%)	450 (47) *	**257 (51)**	**51 (36)**	**142 (48)**
Neoplasms including lymphopoetic system no, (%)	**220 (23)**	**104 (20)**	**47 (33)**	**69 (23)**
Diseases of respiratory system no, (%)	**57 (6)**	**34 (7)**	5 (3)	**18 (6)**
Diseases of the musculosceletal system and connective tissue disease no, (%)	48 (5)	25 (5)	4 (3)	**19 (6)**
External causes of death no, (%)	38 (4)	17 (3)	**10 (7)**	11 (4)
Other diseases no, (%)	137 (15)	72 (14)	25 (18)	40 (13)

* Numbers in bold represent the three most common causes of death in each group.

**Table 3:** Standardized mortality ratios for separate disease entities.

Standardized Mortality Ratios	Rheumatoid Arthritis (M05, M06)	Psoriatic Arthritis (M07)	Ankylosing Spondylitis (M45, M46)	Systemic Lupus Erythematosus (M32)	Sjogren Syndrome (M35.0)	Systemic Sclerosis (M34)	Vasculitis (M30, M31)	Myositis (M33)	Polymyalgia Rheumatica (M35.3)
Total	1.25 (1.14; 1.36)	1.04 (0.81; 1.31)	1.25 (0.88; 1.71)	2.53 (1.59; 3.83)	1.50 (0.98; 2.20)	2.66 (1.49; 4.39)	3.24 (2.59; 4.01)	3.24 (2.59; 4.01)	1.29 (1.07; 1.53)
Women	1.26 (1.13; 1.41)	0.90 (0.56; 1.38)	1.11 (0.48; 2.19)	2.72 (1.64; 4.25)	1.23 (0.72; 1.97)	2.23 (0.82; 4.85)	3.38 (2.42; 4.60)	1.43 (0.30; 4.18)	1.29 (1.03; 1.60)
Men	1.23 (1.05; 1.42)	1.11 (0.83; 1.47)	1.75 (0.36; 5.12)	2.80 (1.03; 6.10)	3.06 (1.40; 5.81)	1.29 (0.87; 1.84)	2.53 (1.16; 4.81)	3.12 (2.28; 4.18)	1.27 (0.93; 1.70)

**Table 4:** Results of the meta-analysis of standardized mortality ratios for rheumatic disease patients.

Disease Entities	Meta-Standardized Mortality Ratio (95% CI) Including Current Study	Heterogeneity, Using I2 (%)	References of Studies *
**Rheumatoid arthritis (M05, M06)**	1.44 (1.32; 1.56)	90.6%	[2,14,16,17,18,19,20,21,22,23,24,25]
**Psoriatic arthritis (M07)**	1.26 (1.08; 1.47)	74.0%	[2,26,27,28,29]
**Ankylosing spondylitis (M46)**	1.59 (1.29; 1.96)	57.1%	[2,30,31]
**Systemic lupus erythematosus (M32)**	2.65 (2.13; 3.28)	95.0%	[2,14,32,33,34,35,36,37,38,39,40,41,42,43,44]
**Sjogren’s syndrome (M35.0)**	1.45 (1.13; 1.86)	87.0%	[14,15,45,46]
**Systemic sclerosis (M34)**	2.55 (1.76;3.68)	95.8%	[2,14,15,47,48,49,50,51,52]
**Vasculitis (M30, M31)**	1.92 (1.44; 2.57)	94.7%	[2,14,15,53,54,55,56,57,58,59,60,61]
**Myositis (M33)**	5.06 (3.67; 6.98)	89.6%	[62,63,64,65]
**Polymyalgia rheumatica (M35.3)**	0.95 (0.52; 1.73)	95.0%	[66]

* the studies included in meta-standardized mortality ratio calculations are provided in the Supplement S10.

## Data Availability

The data presented in this study are available on request from the corresponding author. The data are not publicly available because they contain the identification code for each person included in this study. Data could not be put in any repository because of local ethical restrictions and new rules that came into force on personal data availability since 2017.

## Supplementary Materials

The following are available online at https://www.mdpi.com/article/10.3390/ijerph182312338/s1, Supplement S1: Flowchart of screening and selection of studies on rheumatoid arthritis, Supplement S2: Flowchart of screening and selection of studies on psoriatic arthritis, Supplement S3: Flowchart of screening and selection of studies on ankylosing spondylitis, Supplement S4: Flowchart of screening and selection of studies on systemic lupus erythematosus, Supplement S5: Flowchart of screening and selection of studies on Sjogren’s syndrome, Supplement S6: Flowchart of screening and selection of studies on systemic sclerosis, Supplement S7: Flowchart of screening and selection of studies on systemic vasculitis or vasculopathies in general or in separate subtypes, Supplement S8: Flowchart of screening and selection of studies on dermatomyositis or polymyositis, Supplement S9: Flowchart of screening and selection of studies on polymyalgia rheumatica, Supplement S10: The studies included in meta-standardized mortality ratio calculation.
