# Supplementary material for: Mortality in Inflammatory Rheumatic Diseases: Lithuanian National Registry Data and Systematic Review
Source: Int J Environ Res Public Health. 2021 Nov 24;18(23):12338. doi: 10.3390/ijerph182312338 (PMC8656671; doi:10.3390/ijerph182312338)
Supplement: Supplementary file 1 [file ijerph-18-12338-s001.zip › ijerph-1471698-supplementary.pdf]

SUPPLEMENTS

Supplement S1. Flowchart of screening and selection of studies on rheumatoid arthritis.

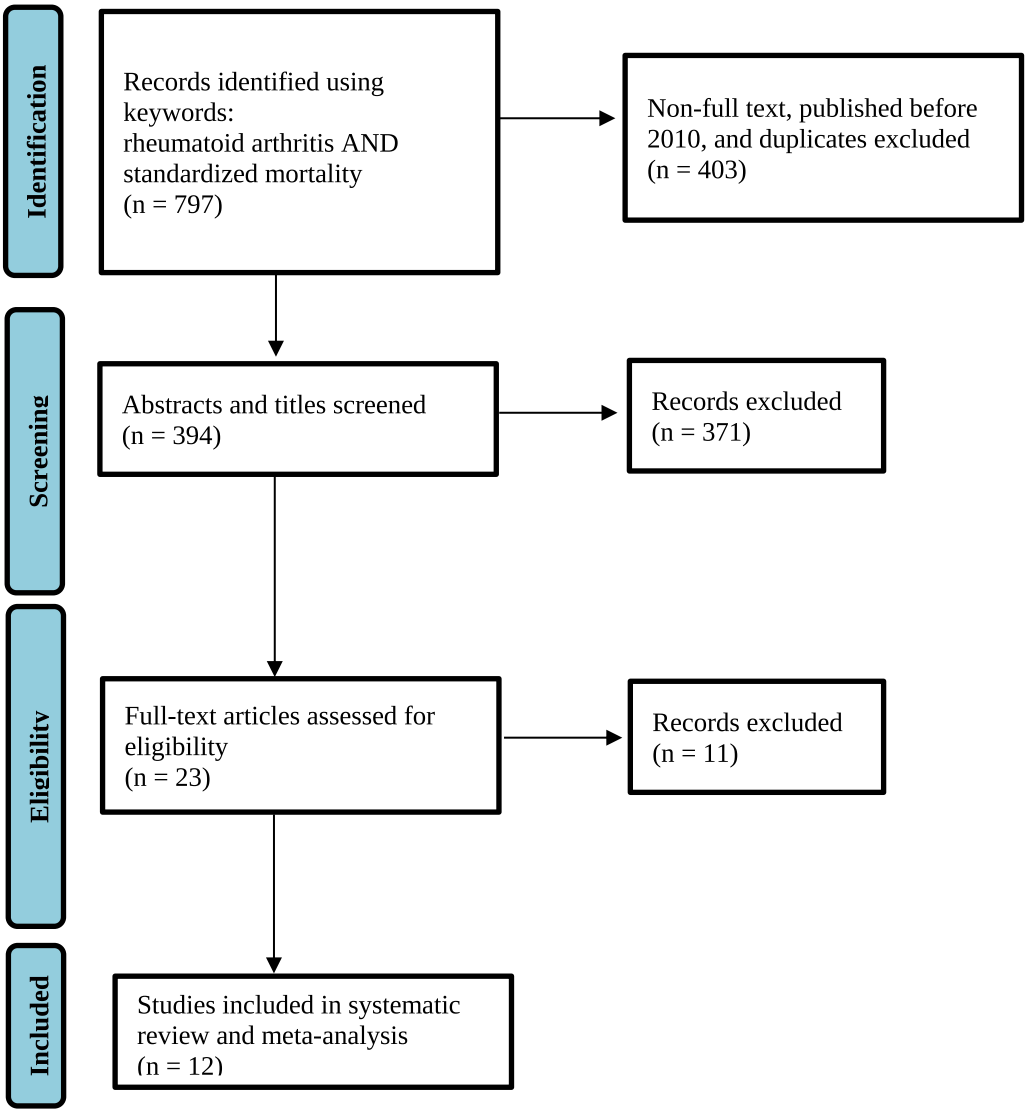

Supplement S2. Flowchart of screening and selection of studies on psoriatic arthritis.

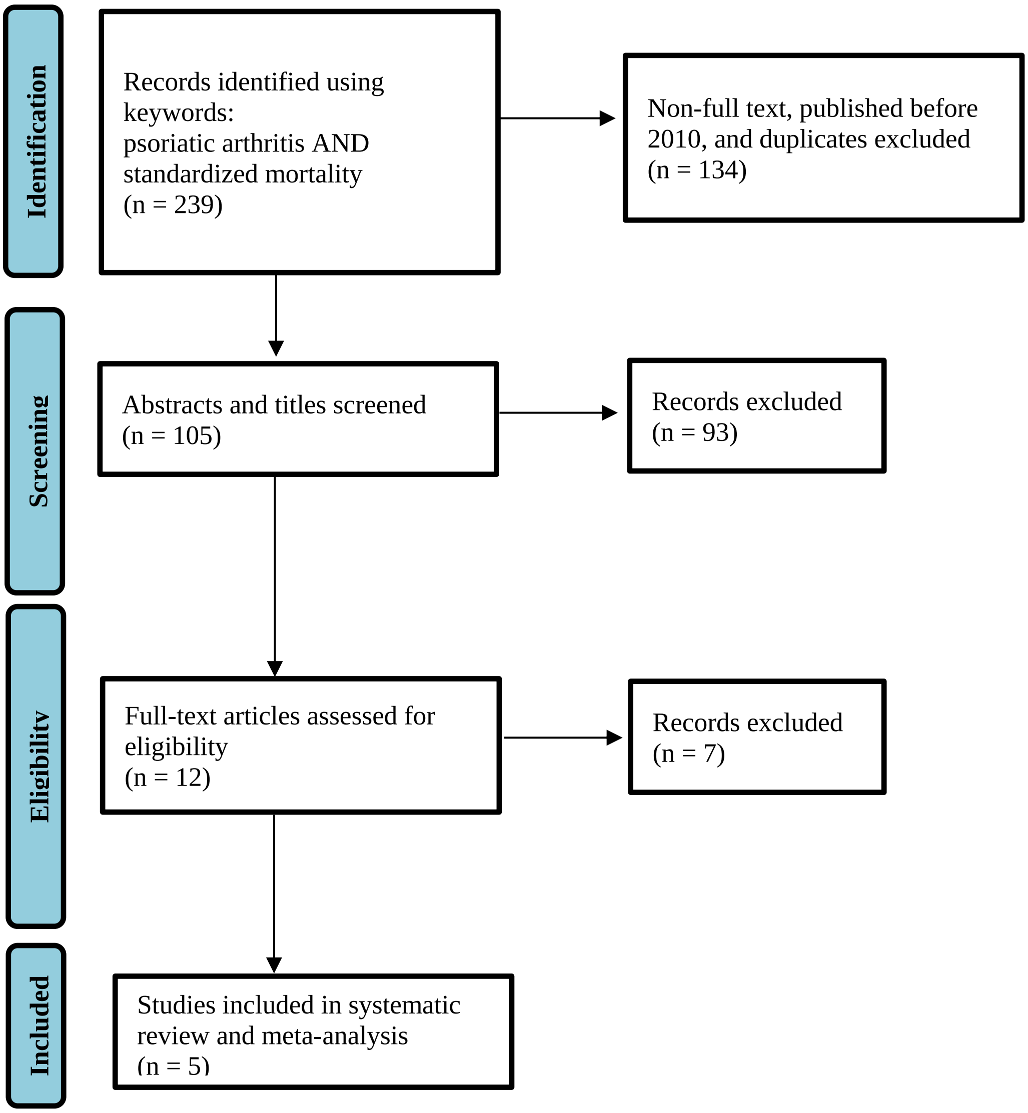

Supplement S3. Flowchart of screening and selection of studies on ankylosing spondylitis.

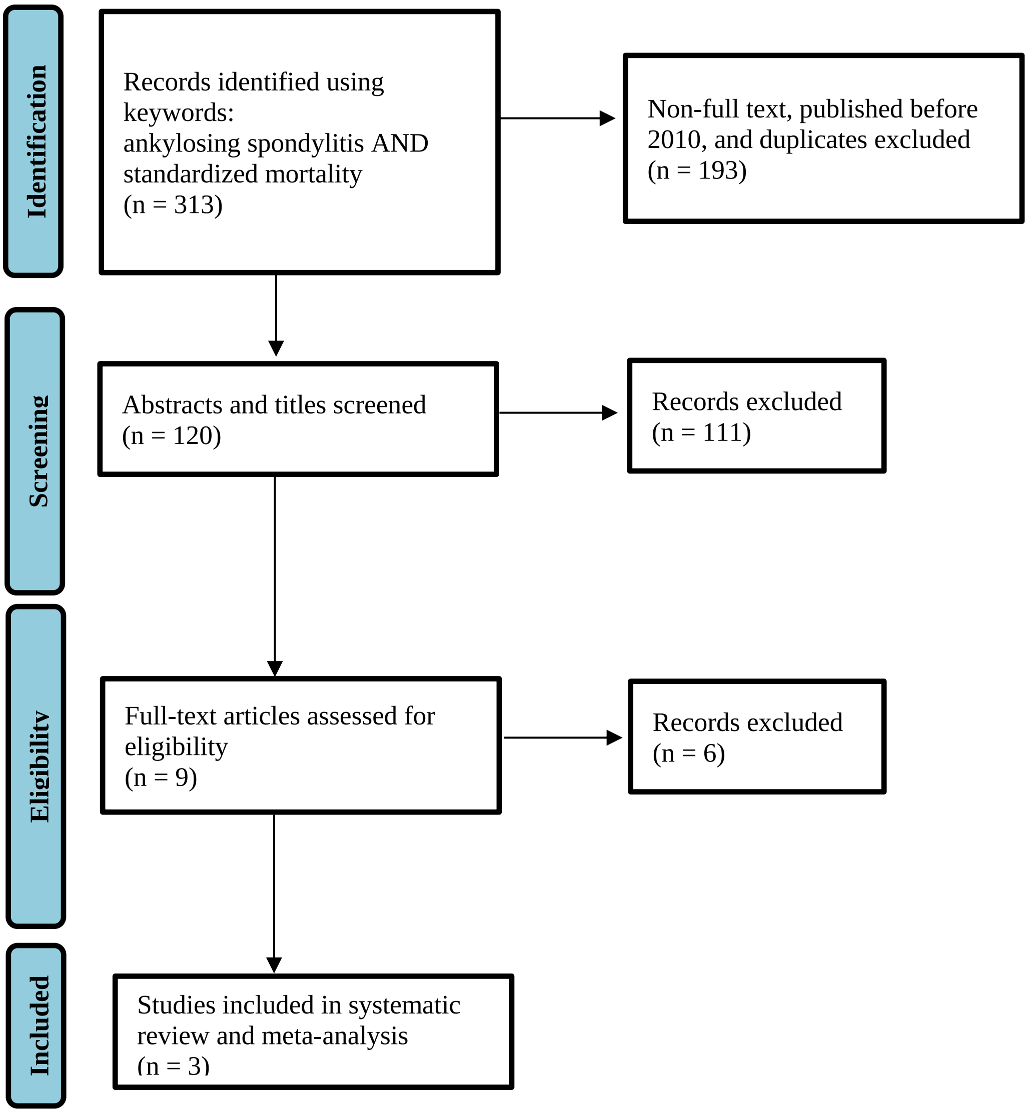

Supplement S4. Flowchart of screening and selection of studies on systemic lupus erythematosus.

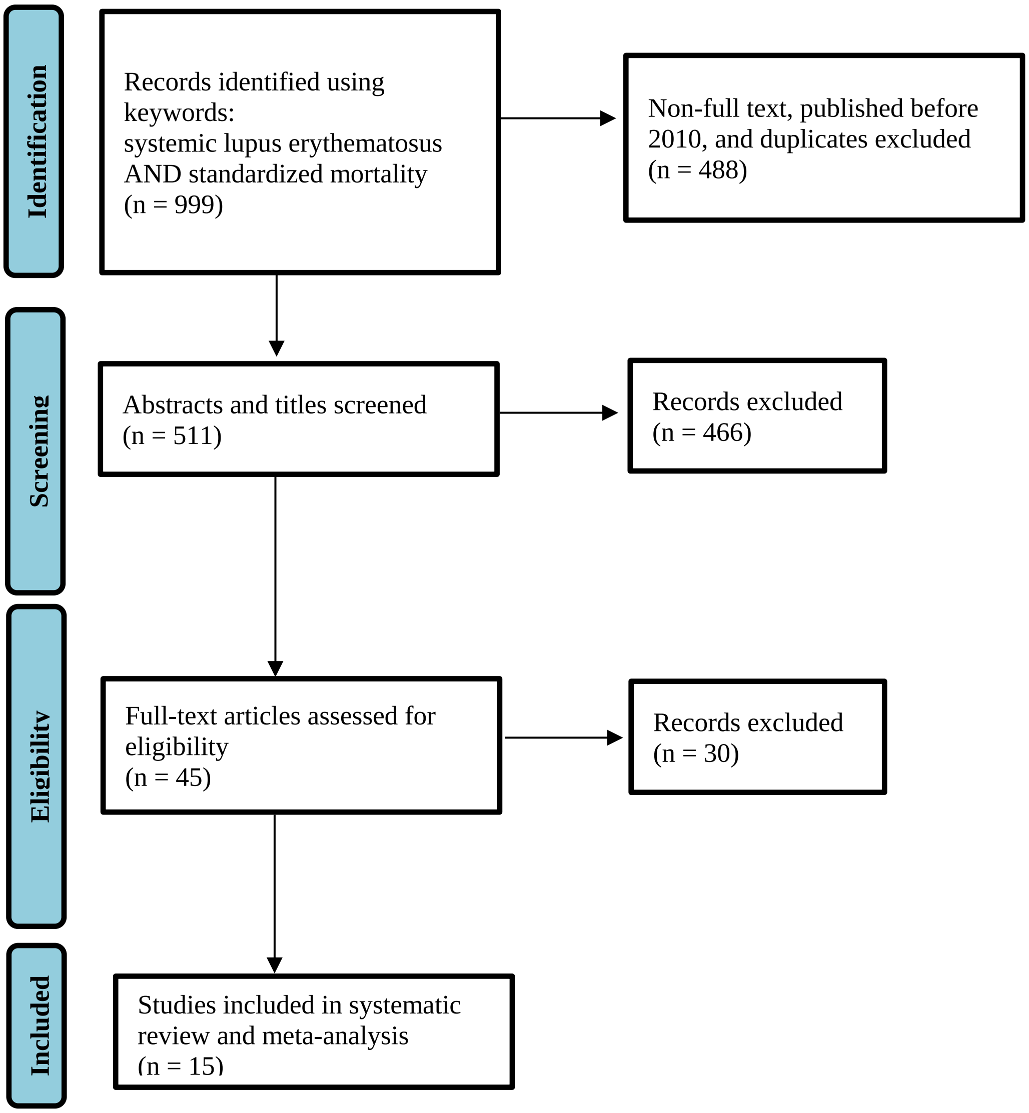

Supplement S5. Flowchart of screening and selection of studies on Sjogren's syndrome.

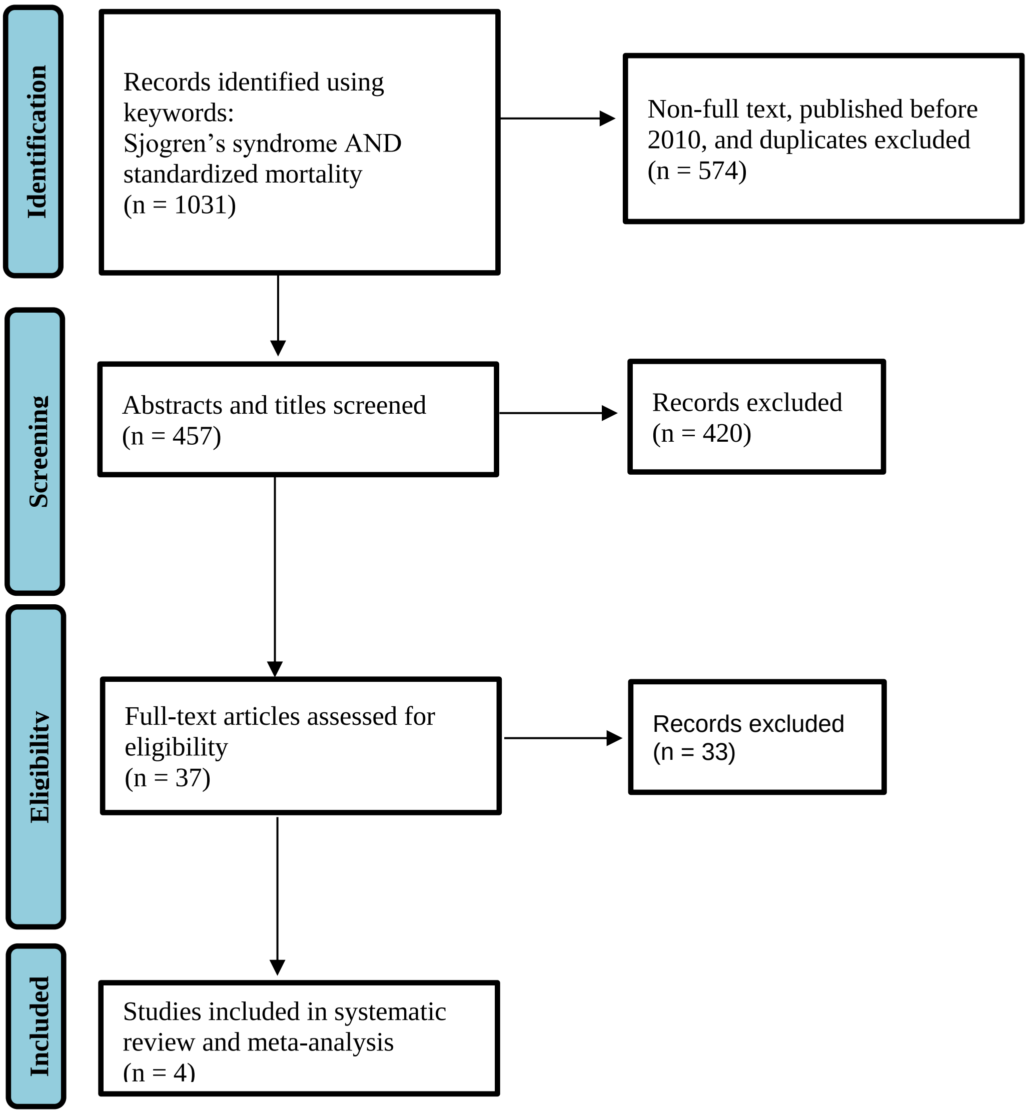

Supplement S6. Flowchart of screening and selection of studies on systemic sclerosis.

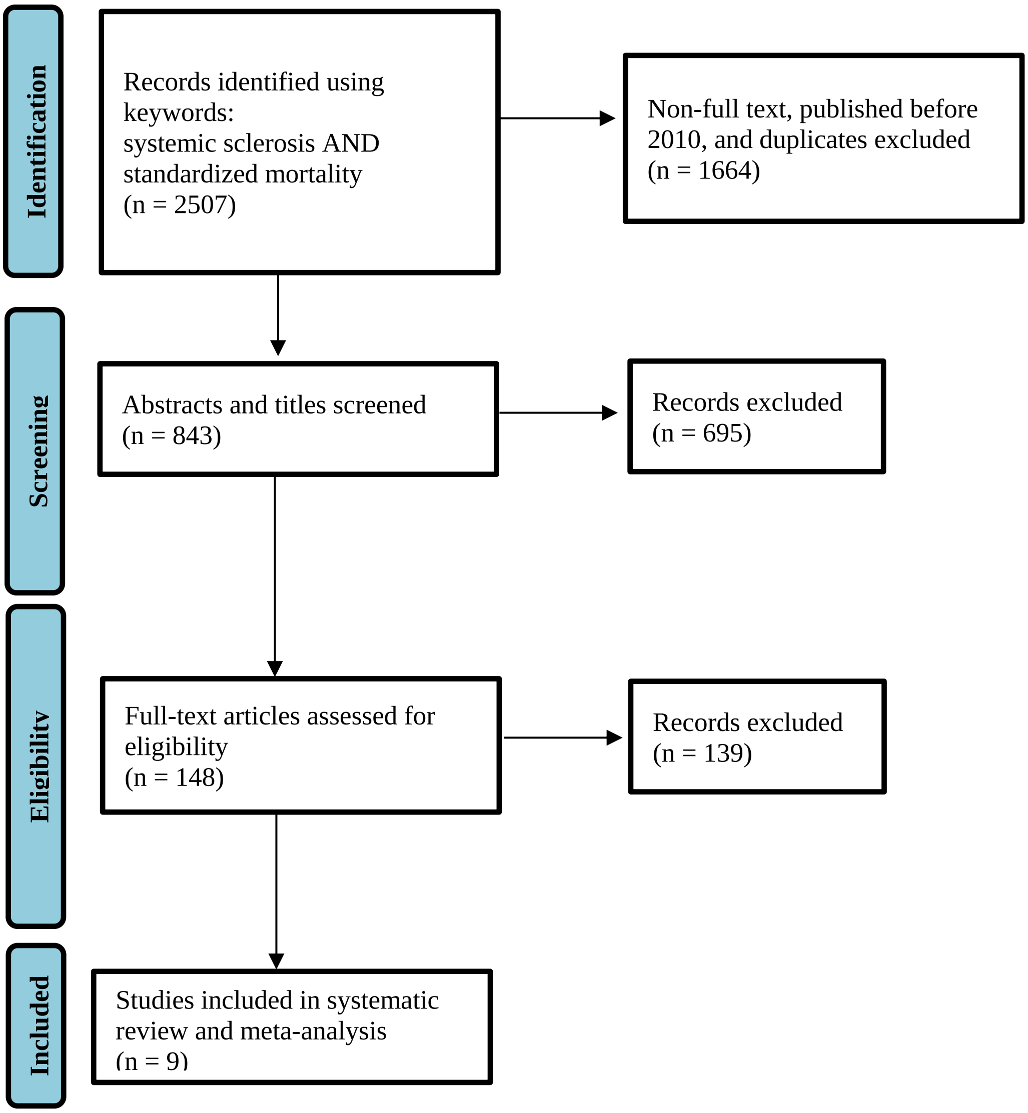

Supplement S7. Flowchart of screening and selection of studies on systemic vasculitis or vasculopathies in general or in separate subtypes.

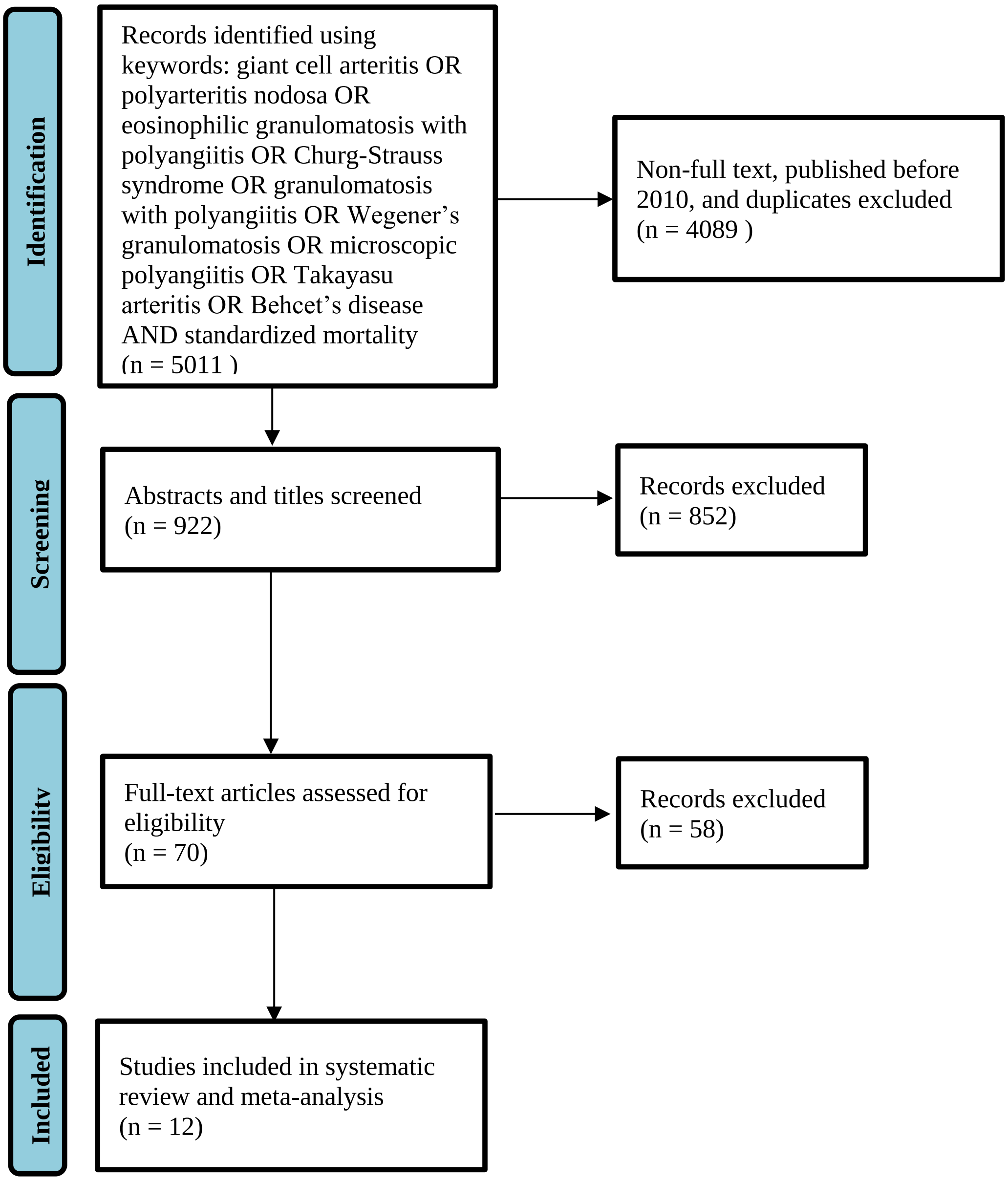

Supplement S8. Flowchart of screening and selection of studies on dermatomyositis or polymyositis.

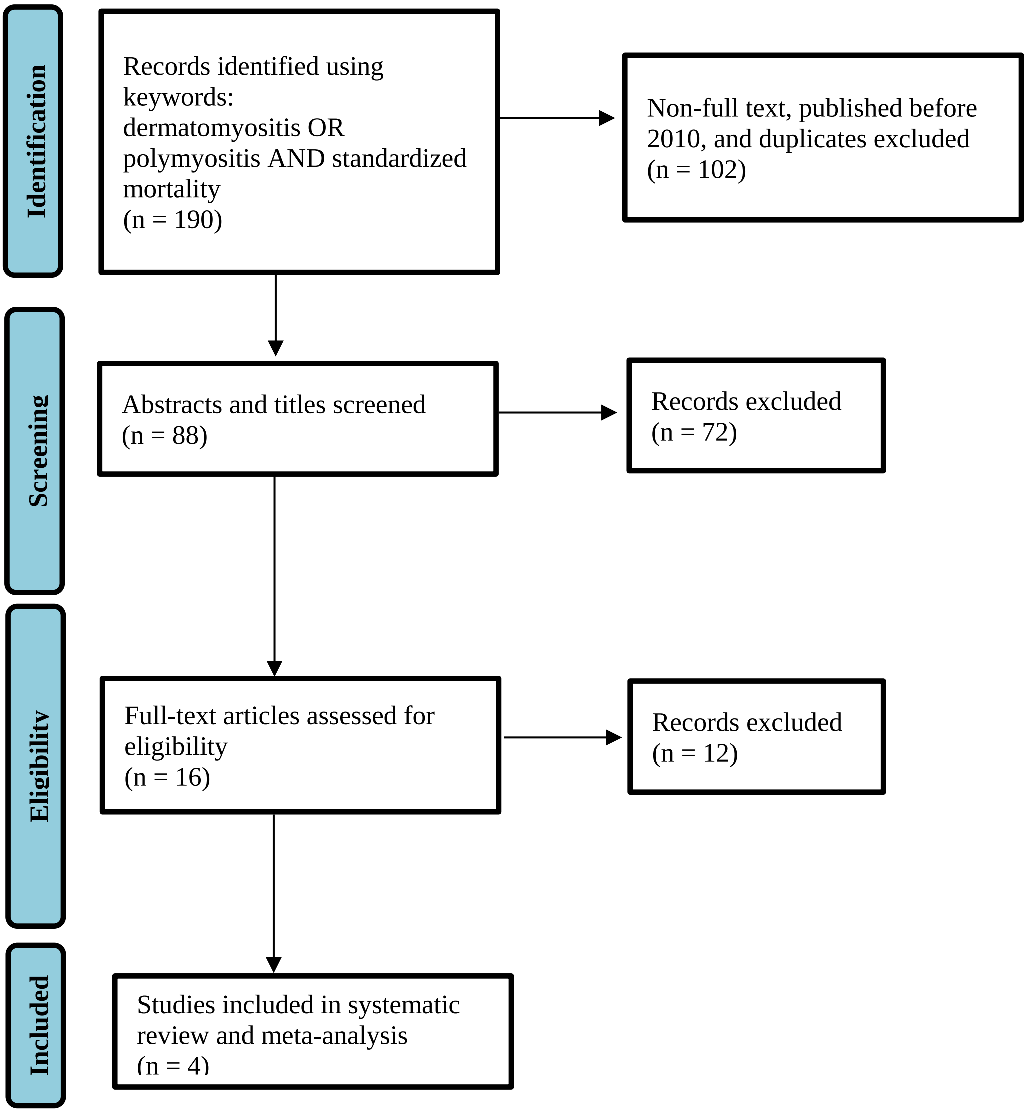

Supplement S9. Flowchart of screening and selection of studies on polymyalgia rheumatica.

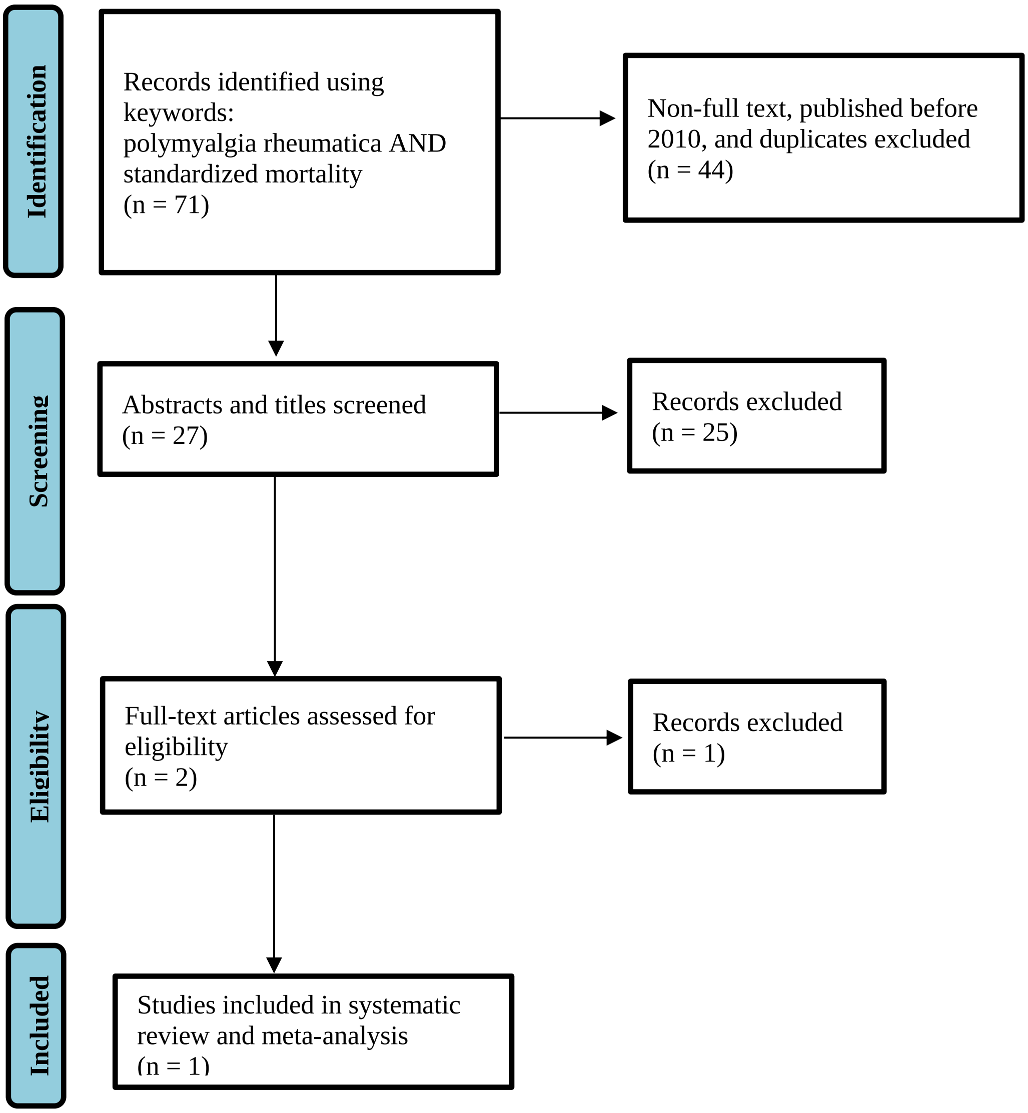

Suppplement S10. The studies included in meta-standardized mortality ratio calculation.

	**Author, Year**	**No of Patients**	**Number of Deaths**	**SMR (95%PI)**
**Rheumatoid arthritis (M05, M06)**	Current study	6008	509	1,3 (1,19;1,41)
	Ometto F. et al, 2018 [16]	16098	2142	1,42 (1,36;1,48)
	Pedersen J. K. et al, 2018 [17]	509	200	1,04 (0,9; 1,19)
	Abasolo et al L., 2016 [18]	2271	431	1,89 (1,72; 2,08)
	van den Hoek J. et al, 2017 [19]	1213	540	1,54 (1,41; 1,67)
	Listing J. et al, 2015 [20]	8908	463	1,49 (1,36; 1,63)
	Humphreys J.H. et al, 2014 [21]	1419	346	1,22 (1,07; 1,4)
	Kuo C. et al, 2013 [22]	15967	985	1,25 (1,18; 1,33)
	Yu K.H. et al., 2013 [14]	1390	131	1,6 (1,3; 1,9)
	Lassere M.N. et al, 2013 [23]	608	251	1,65 (1,44; 1,85)
	Kapetanovic M.C. et al, 2011 [24]	183	69	1,23 (0,97; 1,55)
	Mok C.C. et al, 2011 [2]	8367	1289	1,68 (1,59; 1,77)
	Troelsen C.C. et al, 2010 [25]	229	163	1,5 (1,2; 1,9)
**Psoriatic arthritis (M07)**	Current study	1824	71	1.04 (0.81;1.31)
	Colaco K. et al, 2021 [26]	15430	221	1,34 (1,16; 1,52)
	Lee M.S. et al, 2017 [27]	9572	682	1,47 (1,36; 1,58)
	Juneblad K. et al, 2016 [28]	464	44	1,22 (0,89; 1,63)
	Mok C.C. et al, 2011 [2]	778	51	1,59 (1,16; 2,03)
	Buckley C. et al, 2010 [29]	453	37	0,82 (0,58; 1,13)
**Ankylosing spondylitis (M45,M46)**	Current study	1465	71	1.25 (0.88;1.71)
	Wright K.A. et al, 2015 [30]	86	3	0,72 (0,15; 2,09)
	Bakland G. et al, 2011 [31]	677	98	1,61 (1,29; 1,93)
	Mok C.C. et al, 2011 [2]	2154	197	1,87 (1,61; 2,13)
**Systemic lupus erythematosus (M32)**	Current study	291	22	2.53 (1.59;3.83)
	Kedves M. et al, 2020 [32]	4503	306	1,63 (1,43; 1,83)
	Lim S.S. et al, 2019 [33]	1335	400	3,12 (2,83; 3,44)
	Tselios K. et al, 2019 [34]	1732	249	4,1 (3,6; 4,6)
	Reppe Moe S.E. et al, 2019 [35]	325	56	2,1 (1,2; 3,4)
	Ingvarsson R.F. et al, 2019 [36]	175	60	2,5 (1,9; 3,3)
	Mu L. et al, 2018 [37]	911	45	3,2 (2,4; 4,0)
	Joo Y.B. et al, 2016 [38]	979	44	2,89 (2,10; 3,88)
	Ambrose N. et al, 2016 [39]	511	76	3,1 (2,6; 3,9)
	Yee C.S. et al, 2015 [40]	382	37	2,0 (1,5; 2,8)
	Elfving P. et al, 2014 [41]	566	30	1,48 (1,01; 2,12)
	Yu K.H. et al, 2013 [14]	671	41	2,9 (2,1; 4,0)
	Voss A. et al, 2013 [42]	215	38	2,2 (1,6; 3,2)
	Gustafsson J.T. et al, 2012 [43]	208	42	2,4 (1,7; 3,0)
	Mok C.C. et al, 2011 [2]	5243	514	5,25 (4,79; 5,70)
	Hersh A.O. et al, 2010 [44]	957	72	2,5 (2,0; 3,2)
**Sjogren's syndrome (M35.0)**	Current study	196	18	1.50 (0.98;2.20)
	Yazisiz V. et al, 2020 [45]	372	33	2,11 (1,39; 2,83)
	Garen T. et al, 2019 [15]	336	42	1,1 (1,0; 1,1)
	Kim H. J. et al, 2017 [46]	5981	126	1,61 (1,34; 1,91)
	Yu K. H. et al, 2013 [14]	855	44	1,3 (1,0; 1,7)

**Systemic sclerosis (M34)**	Current study	84	15	2.66 (1.49;4.39)
	Ciaffi J. et al, 2021 [47]	11558	322	2,8 (1,9; 3,8)
	Garen T. et al, 2019 [15]	454	104	3,1 (2,5; 3,8)
	Ooi C. et al, 2018 [48]	132	20	2,59 (1,67; 4,01)
	Butt S. A. et al, 2018 [49]	2778	156	5,7 (4,7;6,4)
	Yu K. H. et al, 2013 [14]	118	9	2,2 (1,2; 4,2)
	Strickland G. et al, 2013 [50]	204	53	1,34 (1,0; 1,75)
	Mok C. C. et al, 2011 [2]	449	110	3,94 (3,20; 4,68)
	Hissaria P. et al, 2011 [51]	786	331	1,46 (1,28; 1,69)
	Perez-Bocanegra C. et al, 2010 [52]	319	24	1,9 (1,5; 2,3)
**Systemic vasculitis or vasculopathies in general or in separate subtypes (M30;M31)**	Current study	345	74	3.24 (2.59;4.01)
	Garen T. et al, 2019 (ANCA vasculitis) [15]	206	31	1,5 (1,0;2,1)
	Garen T. et al, 2019 (TAK) [15]	108	9	2,5 (1,1; 4,9)
	Garen T. et al, 2019 (GCA) [15]	189	22	0,5 (0,3; 0,7)
	Mirouse A. et al, 2019 (TAK) [53]	318	16	2,73 (1,69; 4,22)
	Park S. J. et al, 2017 (TAK) [54]	612	64	3,1 (2,4; 4,0)
	Schirmer J. H. et al, 2016 (MP) [55]	144	21	1,4 (0,91; 2,07)
	Kermani T.A. et al, 2014 (GCA) [56]	204	154	1,0 (0,9; 1,2)
	Schmidt J, et al, 2013 (TAK) [57]	126	6	3,0 (1,0; 8,9)
	Yu K. H. et al, 2013 (SV) [14]	97	5	2,6 (1,1; 6,2)
	Yu K. H. et al, 2013 (BD) [14]	64	3	3,7 (1,2; 11,6)
	Mok C. C. et al, 2011 (SV) [2]	1636	325	2,64 (2,36; 2,93)
	Ninan J. et al, 2011 (GCA) [58]	225	71	0,99 (0,77; 1,25)
	Holle J. U. et al, 2011 (WG) [59]	445	43	1,58 (1,14; 2,13)
	Takala JH. et al, 2010 (WG) [60]	492	203	3,43 (2,98; 3,94)
	Saadon D. et al, 2010 (BD) [61]	817	41	2,10 (1,57; 2,87)
**Myositis, polymyositis or dermatomyositis (M33)**	Current study	**74**	9	3.24 (2.59;4.01)
	Kridin K. et al, 2020 (DM) [62]	82	30	7,2 (5,0; 10,3)
	Kridin K. et al, 2020 (PM) [62]	84	17	7,7 (4,8; 12,3)
	Yang X. et al, 2020 (DM) [63]	226	55	9,0 (6,8; 11,2)
	Yang X. et al, 2020 (PM) [63]	85	13	6,0 (3,5; 8,5)
	Dobloug G.C. et al, 2015 (DM) [64]	128	26	2,6 (1,8; 3,9)
	Dobloug G.C. et al, 2015 (PM) [64]	98	30	2,4 (1,7; 3,3)
	Kuo C.F. et al, 2011 (DM) [65]	803	146	7,68 (6,41; 9,01)
	Kuo C.F. et al, 2011 (PM) [65]	500	90	5,29 (4,28; 6,48)
**Polymyalgia rheumatica (M35.3)**	Current study	728	102	1.29 (1.07;1.53)
	Raheel S. et al, 2017 [66]	377	107	0,70 (0,57; 0,85)

ANCA – anti-neutrophil cytoplasmic antibody, TAK – Takayasu arteritis, GCA – giant cell arteritis, MP – microscopic polyangiitis, SV – systemic vasculitis, BD – Behcet’s disease, WG – Wegener’s granulomatosis, DM – dermatomyositis, PM – polymyositis.
